# Accurate modeling of replication rates in genome-wide association studies by accounting for Winner’s Curse and study-specific heterogeneity

**DOI:** 10.1093/g3journal/jkac261

**Published:** 2022-10-17

**Authors:** Jennifer Zou, Jinjing Zhou, Sarah Faller, Robert P Brown, Sriram S Sankararaman, Eleazar Eskin

**Affiliations:** Computer Science Department, University of California, Los Angeles, CA 90095, USA; Computer Science Department, University of California, Los Angeles, CA 90095, USA; Computer Science Department, Duke University, Durham, NC 27708, USA; Computer Science Department, University of California, Los Angeles, CA 90095, USA; Computer Science Department, University of California, Los Angeles, CA 90095, USA; Computer Science Department, University of California, Los Angeles, CA 90095, USA; Department of Human Genetics, University of California, Los Angeles, CA 90095, USA

**Keywords:** genome-wide association study (GWAS), Winner’s Curse, confounding, replication

## Abstract

Genome-wide association studies (GWAS) have identified thousands of genetic variants associated with complex human traits, but only a fraction of variants identified in discovery studies achieve significance in replication studies. Replication in genome-wide association studies has been well-studied in the context of Winner’s Curse, which is the inflation of effect size estimates for significant variants due to statistical chance. However, Winner’s Curse is often not sufficient to explain lack of replication. Another reason why studies fail to replicate is that there are fundamental differences between the discovery and replication studies. A confounding factor can create the appearance of a significant finding while actually being an artifact that will not replicate in future studies. We propose a statistical framework that utilizes genome-wide association studies and replication studies to jointly model Winner’s Curse and study-specific heterogeneity due to confounding factors. We apply this framework to 100 genome-wide association studies from the Human Genome-Wide Association Studies Catalog and observe that there is a large range in the level of estimated confounding. We demonstrate how this framework can be used to distinguish when studies fail to replicate due to statistical noise and when they fail due to confounding.

## Introduction

Replication is a gold standard in scientific discovery. Consensus emerges when a result has been replicated repeatedly by multiple researchers. Recently, a vigorous discussion has emerged of how often replication of an initial study fails across all fields of science, including genomics (Ioannidis 2005, 2016; Patil *et al.* 2015; Palmer and Pe’er 2017; Marigorta *et al.* 2018).

Genome-wide association studies (GWAS) are an ideal model to study replication because there are a large number of GWAS data sets with replication studies publicly available. GWAS replication studies are typically conducted in an independent cohort using only the variants that were significant in the initial (“discovery”) study. In the National Human Genome Research Institute Catalog of Published GWAS, thousands of genetic variants have been associated with complex human traits but not all associated variants achieve significance in replication studies (Greene *et al.* 2009; Welter *et al.* 2014; Palmer and Pe’er 2017; Marigorta *et al.* 2018).

There are several reasons why associations do not replicate. The first is simply statistical. It is possible that the association is not observed in the replication study by chance. However, if the *P*-value from the original finding is highly significant and the replication studies have similar experimental designs, this scenario is unlikely. A second reason why studies can fail to replicate is Winner’s Curse (WC), which is the inflation of effect size estimates for significant variants in a study due to statistical chance. This phenomenon occurs because the reported findings are a small fraction of many possible findings. In the case of GWAS, the significant associations are discovered after examining millions of variants and pass a stringent genome-wide significance threshold. This can result in inflated effect size estimates of significant variants in a study, especially when studies have low power (Xu 2003). WC has been studied extensively in GWAS, and multiple methods have been proposed to correct for its effects (Xiao and Boehnke 2009, 2011; Zhong and Prentice 2010; Sun *et al.* 2011; Palmer and Pe’er 2017). However, WC is often not sufficient to explain lack of replication. A third reason why studies fail to replicate is that there are fundamental differences between the discovery and the replication study or “study-specific heterogeneity.” An effect present in one study but not present in other studies can create the appearance of a significant finding that is not replicated in future studies (Kang *et al.* 2008). For example, differences in minor allele frequency of variants due to ancestry between 2 studies could result in differences in effect sizes and lack of replication (Palmer and Pe’er 2017). This can either occur because of an underlying biological difference or a technical difference between the 2 studies. We refer to the cause of these differences as confounders.

Current methods for modeling confounders fall into 2 broad categories. The first class of methods attempts to model the effect of confounders before the association statistic is calculated in order to remove their effects from the association statistic (Patterson *et al.* 2006; Stegle *et al.* 2012). While these methods are widely used, they have several fundamental limitations. Methods that account for known covariates may not correct for all potential confounders. Confounding correction methods that use unsupervised learning to learn principal components or other global patterns in the data can incorrectly model the true signal as a confounder, which would remove true biological signal from the data (Price *et al.* 2006; Joo *et al.* 2014). Similarly, when using unsupervised methods, it is unclear when there is residual confounding that remains in the data. The second class of methods attempts to directly adjust association statistics by a constant factor to remove inflation (Devlin and Roeder 1999; Bulik-Sullivan *et al.* 2015). An example of such a method is genomic control (Devlin and Roeder 1999). In genomic control, there is an assumption that relatively few variants affect the trait. The implication of this assumption is that if the association statistics are ranked, then the variant corresponding to the median statistic will not affect the trait, and the value of this statistic (*λ_GC_*) will represent only the effect of the confounders. Genomic control scales all of the association statistics using *λ_GC_*. Recently, it has been observed that due to polygenicity and linkage disequilibrium (LD) structure in the genome, the majority of variants (including the one corresponding to the median statistic) either affect the trait or are correlated with variants that affect the trait. This breaks the genomic control assumption. While LD-score regression has been shown to distinguish polygenicity and confounding (Bulik-Sullivan *et al.* 2015), it has been shown that this approach can also result in inflated SNP-based heritability estimates under strong stratification (de Vlaming *et al.* 2017).

In this paper, we present a novel approach for characterizing study-specific heterogeneity due to confounders using replication studies. The key insight in our approach is that we can use replications to estimate the effects of confounders and then account for their effects. Since replication studies are performed on the same phenotype, polygenicity should be the same between the 2 studies. Because we are using replication studies, we do not need to assume that the variant corresponding to the median statistic will not affect the trait to estimate confounding, like in genomic control. Furthermore, we can apply our approach in combination with traditional techniques like linear mixed models and regressing out the effect of covariates that are applied before computing association statistics. Our approach can be used to model any residual confounding effects after application of these methods.

In our framework, we perform a bivariate analysis between the z-scores from the discovery study and the z-scores from the replication study, while modeling the effects of both WC and study-specific confounders. We show through simulations that we can accurately estimate the contribution of study-specific confounders on a study and use this estimate to explain observed patterns of replication. We apply this framework to 100 GWAS from the Human GWAS Catalog and observe that there is a large range in the level of confounding observed across GWAS. We show that our estimate levels of confounding correlates well with observed patterns of replication and demonstrate how this can be used to distinguish when studies fail to replicate due to statistical noise and when they fail due to confounding.

## Methods

### GWAS overview

In GWAS, an association study is performed between each genetic variant and the phenotype using a linear model. The effect size of each variant (*k*) is determined by estimating the maximum likelihood parameters of Equation (1), where *y_j_* is the normalized phenotype for individual *j*, *x_kj_* is the standardized genotype of variant *k* in individual *j*, *β_k_* is the effect size of the variant *k*, *e_j_* is the error, and *N* is the number of individuals.

(1) $$y_{j}=\beta_{k}x_{kj}+e_{j}.$$

In vector notation, Equation (1) becomes Equation (2), where *y* is a vector of phenotypes for all individuals, *β* is a vector of betas for all variants, *X* is a matrix of normalized genotypes for all individuals and variants, and **e** is a vector of errors.

(2) y=βX+e.

The resulting maximum likelihood estimates (MLE) are $\hat{\mu }=\frac{1}{N}1^{T}y$ and $\hat{\beta_{k}}=\frac{X_{k}^{T}y}{N}$ (Eskin 2015). The residuals $\hat{e}=y-\hat{\mu }1-\hat{\beta_{k}}X_{k}$ can be used to estimate the standard error $\hat{\sigma_{e}}=\sqrt{\frac{{\hat{e}}^{T}\hat{e}}{N-2}}$. The standard error of the estimator is $\hat{\sigma_{\beta_{k}}}=\frac{\hat{\sigma_{e}}}{\sqrt{N}}$. The association statistic $s_{k}=\frac{\hat{\beta_{k}}}{\hat{\sigma_{e}}}\sqrt{N}$ follows a Student’s *t*-distribution. For large samples, it is asymptotically approximated to a normal distribution (Equation 3;Eskin 2015).

(3) $$s_{k}∼N\left(\frac{\beta_{k}}{\sigma_{e}}\sqrt{N},1\right).$$

Under the null hypothesis, *S_k_* will follow the standard normal distribution, which can be used to compute the significance of association. In the standard GWAS framework, we assume that the standardized effect size is caused by a true genetic effect $\lambda =\frac{\beta_{k}}{\sigma_{e}}$. Thus, Equation (3) can be rewritten as the following:

(4) $$s_{k}|\lambda ∼N\left(\lambda \sqrt{N},1\right).$$

### Correcting GWAS statistics for WC

Let *N*_1_ be the sample size of the discovery study and *N*_2_ be the sample size of the replication study. Given Equation (3), we can write the distributions of association statistics for a discovery study and a replication study as $s_{k}^{\left(1\right)}|\lambda ∼N\left(\lambda \sqrt{N_{1}},1\right)$ and $s_{k}^{\left(2\right)}|\lambda ∼N\left(\lambda \sqrt{N_{2}},1\right)$, respectively.

We assume that *λ* is the same across multiple studies on the same trait. We define the prior distribution of *λ* as $\lambda ∼N(0,\sigma_{g}^{2})$, where $\sigma_{g}^{2}$ is the variance in the true effect size. We combine Equation (4) and the prior distribution on *λ* and integrate over all possible values to *λ* to obtain the posterior distributions of $s_{k}^{\left(1\right)}$ and $s_{k}^{\left(2\right)}$, which are also normally distributed.

(5) $$s_{k}^{\left(1\right)}∼N(0,N_{1}\sigma_{g}^{2}+1)$$

(6) $$s_{k}^{\left(2\right)}∼N(0,N_{2}\sigma_{g}^{2}+1).$$

We correct for WC by computing the conditional distribution of the replication statistic ($s_{k}^{\left(2\right)}$) given the discovery statistic ($s_{k}^{\left(1\right)}$). We derive the conditional distribution from the joint distribution as follows.

The covariance between $s_{k}^{\left(1\right)}$ and $s_{k}^{\left(2\right)}$ is computed as follows:

$$\begin{array}{l} cov(s_{k}^{\left(1\right)},s_{k}^{\left(2\right)})=E\left[\left(\lambda \sqrt{N_{1}}-E(\lambda \sqrt{N_{1}}))(\lambda \sqrt{N_{2}}-E(\lambda \sqrt{N_{2}})\right)\right]\\ =E\left[\lambda^{2}\sqrt{N_{1}N_{2}}\right]\\ =\sqrt{N_{1}N_{2}}\sigma_{g}^{2} \end{array}.$$

Therefore, the joint distribution of $s_{k}^{\left(1\right)}$ and $s_{k}^{\left(2\right)}$ is Equation (7).

(7) $$\begin{array}{c} \left(\begin{array}{c} s_{k}^{\left(1\right)}\\ s_{k}^{\left(2\right)} \end{array}\right)∼N\left(\left(\begin{array}{c} 0\\ 0 \end{array}\right),\left(\begin{array}{cc} N_{1}\sigma_{g}^{2}+1 & \sqrt{N_{1}N_{2}}\sigma_{g}^{2}\\ \sqrt{N_{1}N_{2}}\sigma_{g}^{2} & N_{2}\sigma_{g}^{2}+1 \end{array}\right)\right) \end{array}.$$

Conditioning on $s_{k}^{\left(1\right)}$, we obtain Equation (8).

(8) $$(s_{k}^{\left(2\right)}|s_{k}^{\left(1\right)}=x)∼N\left(\frac{\sqrt{N_{1}N_{2}}\sigma_{g}^{2}}{N_{1}\sigma_{g}^{2}+1}x,1+N_{2}\sigma_{g}^{2}-\frac{N_{2}\sigma_{g}^{4}}{N_{1}\sigma_{g}^{2}+1}\right).$$

For each value of $s_{k}^{\left(1\right)}$, the mean of the conditional distribution gives the expected statistic in a replication study, correcting for WC. This distribution can also be used to create a confidence interval on the replication sample statistics.

### Correcting GWAS statistics for WC and confounding

Suppose in addition to study-specific environmental effects, there are also study-specific confounders. We model these confounders in the discovery study and replication study as $\delta^{\left(1\right)}∼N(0,\sigma_{c_{1}}^{2})$ and $\delta^{\left(2\right)}∼N(0,\sigma_{c_{2}}^{2})$, respectively. We decompose the effect size into the sum of a genetic component (*λ*) and a confounding component $\delta^{\left(i\right)}$.

(9) $$s_{k}^{\left(1\right)}|\lambda ∼N\left((\lambda +\delta^{\left(1\right)})\sqrt{N_{1}},1\right)$$

(10) $$s_{k}^{\left(2\right)}|\lambda ∼N\left((\lambda +\delta^{\left(2\right)})\sqrt{N_{2}},1\right).$$

Similar to the case without confounding, the posterior distributions of $s_{k}^{\left(1\right)}$ and $s_{k}^{\left(2\right)}$ are normally distributed (Equations 11 and 12).

(11) $$s_{k}^{\left(1\right)}∼N(0,N_{1}\sigma_{g}^{2}+N_{1}\sigma_{c_{1}}^{2}+1)$$

(12) $$s_{k}^{\left(2\right)}∼N(0,N_{2}\sigma_{g}^{2}+N_{2}\sigma_{c_{2}}^{2}+1).$$

Therefore, the joint distribution is Equation (13).

(13) $$\begin{array}{c} \left(\begin{array}{c} s_{k}^{\left(1\right)}\\ s_{k}^{\left(2\right)} \end{array}\right)∼N\left(\left(\begin{array}{c} 0\\ 0 \end{array}\right),\left(\begin{array}{cc} N_{1}\sigma_{g}^{2}+N_{1}\sigma_{c_{1}}^{2}+1 & \sqrt{N_{1}N_{2}}\sigma_{g}^{2}\\ \sqrt{N_{1}N_{2}}\sigma_{g}^{2} & N_{2}\sigma_{g}^{2}+N_{2}\sigma_{c_{2}}^{2}+1 \end{array}\right)\right) \end{array}.$$

Similar to the WC only model, we can find the expected statistic in a replication study correcting for WC by computing the conditional distribution of the replication statistic ($s_{k}^{\left(2\right)}$) given the discovery statistic ($s_{k}^{\left(1\right)}$) (Equation 14).

(14) $$(s_{k}^{\left(2\right)}|s_{k}^{\left(1\right)}=x)∼N\left(\frac{\sqrt{N_{1}N_{2}}\sigma_{g}^{2}}{N_{1}\sigma_{g}^{2}+N_{1}\sigma_{c_{1}}^{2}+1}x,N_{2}\sigma_{g}^{2}+N_{2}\sigma_{c_{2}}^{2}+1-\frac{N_{1}N_{2}\sigma_{g}^{4}}{N_{1}\sigma_{g}^{2}+N_{1}\sigma_{c_{1}}^{2}+1}\right).$$

### Estimating the variance components from data

The variance in the true genetic effect ($\sigma_{g}^{2}$) and variance in the confounding effects ($\sigma_{c_{1}}^{2},\;\sigma_{c_{2}}^{2}$) are not known a priori. We estimate these parameters from the data by maximizing the log-likelihood of the joint distribution for discovery and replication z-scores (Equation 13). We compute the maximum likelihood estimators of the variance parameters using the Nelder–Mead method implemented in the scipy.optimize package (https://docs.scipy.org/doc/scipy/reference/optimize.minimize-neldermead.html).

Since typically only a subset of the data that were significant in the discovery is observed, we account for missing data by integrating over all possible values. Let the significance threshold of the discovery study be *t* (typically a genome-wide significance threshold, such as 5e−8), and let *z* be the corresponding z-score from a unit normal distribution. We use the joint distribution of the z-scores to compute the probability of a variant not being significant in the discovery study ($1-2P(s^{\left(1\right)}<z)$). If the total number of variants that were tested is *N* and the set of significant variants in the discovery study is A, the negative log-likelihood accounting for missing data is Equation (15). The first term corresponds to the likelihood of variants that were not significant in the discovery study, while the second term corresponds to those that were significant. When all variants are provided for both studies, the first term goes to zero, and Equation (19) is simply the joint likelihood of the 2 studies.

(15) $$\left(N-|A|)(1-2P(s^{\left(1\right)}<z))+\sum_{k\in A}P(s_{k}^{\left(1\right)},s_{k}^{\left(2\right)}\right).$$

An implementation of our framework is publicly available (https://github.com/jzou1115/wcrep). Since the negative log likelihood function is convex, run time for parameter estimation is negligible.

### Computing expected replication rates

We computed the expected replication rate under each model using 2 conditional distributions $\left(s_{k}^{\left(2\right)}|s_{k}^{\left(1\right)}\right)$ (Equations 14 and 8).

Let A be the set of variants found to be significant in the discovery study. We used a nominal threshold of 0.05 for the replication study. Let *z* be the z-score threshold corresponding to *t*. For a genetic variant *k* with association statistic $s_{k}^{\left(1\right)}=x$ in a discovery study, the probability of replication is $Pr\left(\mathrm{abs}(s_{k}^{\left(2\right)})>\mathrm{abs}(z)|s_{k}^{\left(1\right)}=x\right)$. We defined the expected replication rate for a study (*r*) as the average probability of replication for variants significant in the discovery study (Equation 16).

(16) $$r=\frac{1}{\left|A\right|}\sum_{s_{k}\in A}P\left(\mathrm{abs}(s_{k}^{\left(2\right)})>z|s_{k}^{\left(1\right)}=x\right).$$

We used the marginal distribution of the discovery summary statistics (Equation 9) to compute the proportion of variance explained by genetics and confounding.

We computed the variance explained by genetics *p_g_* as

(17) $$p_{g}=\frac{N_{1}\sigma_{g}^{2}}{N_{1}\sigma_{g}^{2}+N_{1}\sigma_{c1}^{2}}=\frac{\sigma_{g}^{2}}{\sigma_{g}^{2}+\sigma_{c1}^{2}}.$$

We computed the variance explained by confounding in the discovery study $p_{c1}$ as

(18) $$p_{c1}=\frac{N_{1}\sigma_{c1}^{2}}{N_{1}\sigma_{g}^{2}+N_{1}\sigma_{c1}^{2}}=\frac{\sigma_{c1}^{2}}{\sigma_{g}^{2}+\sigma_{c1}^{2}}.$$

### Data generating model

For all simulations, we fixed the sample size of the discovery study ($N_{1}=2,000$) and the sample size of the replication study ($N_{2}=1,000$).

For each simulation, We fixed the variance parameters to be one of 4 values $\sigma_{g},\sigma_{c1},\sigma_{c2}\in [.018,.022,.026,.03]$. These values were selected to obtain a realistic range of numbers of significant variants in the discovery study (<1% of the variants). We simulated summary statistics for 1 million SNPs using the following procedure. For each SNP *k*, we drew true genetic ($\lambda_{k}∼N(0,\sigma_{g}^{2})$) and confounding effects ($\delta_{k}^{\left(1\right)}∼N(0,\sigma_{c_{1}}^{2})$ for the discovery study and $\delta_{k}^{\left(2\right)}∼N(0,\sigma_{c_{2}}^{2})$ for the replication study). We assume that the effect *λ* in Equation (4) is the sum of a genetic effect and a study-specific confounding effect. Then, we simulated the z-scores for SNP *k* as the sum of the genetic effect and the study-specific confounding effect, scaled for the sample size of the study.

(19) $$s_{k}^{\left(1\right)}∼N\left(\sqrt{N_{1}}(\lambda_{k}+\delta_{k}^{\left(1\right)}),1\right)$$

(20) $$s_{k}^{\left(2\right)}∼N\left(\sqrt{N_{2}}(\lambda_{k}+\delta_{k}^{\left(2\right)}),1\right).$$

While we fix the sample sizes for each study, we vary the effect sizes of the genetic effect and study-specific confounding effects. Since the sample size is multiplied by the effect sizes, this is equivalent to varying the effect sizes.

We simulated data for every possible combination of parameter values ($4^{3}=64$ combinations) and repeated this procedure 1,000 times for a total of 64,000 simulations. For all simulations, we used a Bonferonni corrected threshold of 5e−8 to identify SNPs significant in the discovery study. The observed replication rate was computed as the fraction of variants significant in the discovery study that met a nominal threshold of 0.05 in the replication study.

We used these simulations to assess the accuracy of our MLE of the parameters and the expected replication rate under the models under 2 scenarios: (1) using complete data and (2) using incomplete data. When using complete data, we used the z-scores for all 1 million variants simulated to estimate the variance components. When using incomplete data, we used z-scores for only the variants that were significant in the discovery study.

In order to compare our WC model to previous methods, we generated a second set of simulations. These simulations were identical to the previous set of simulations, except that we fixed the variance in the study-specific confounders to be zero. Thus, we simulated the z-scores for each SNP *k* as

(21) $$s_{k}^{\left(1\right)}∼N\left(\sqrt{N_{1}}\lambda_{k},1\right)$$

(22) $$s_{k}^{\left(2\right)}∼N\left(\sqrt{N_{2}}\lambda_{k},1\right).$$

## Results

### Method overview

The main goal of this framework is to account for WC and study-specific confounding in discovery and replication GWAS of the same phenotype. We compare this model to a naive model that only accounts for WC. We introduce these 2 models without accounting for difference in sample size for clarity, but we relax this constraint in the *Methods* section.

**Fig. 1.**
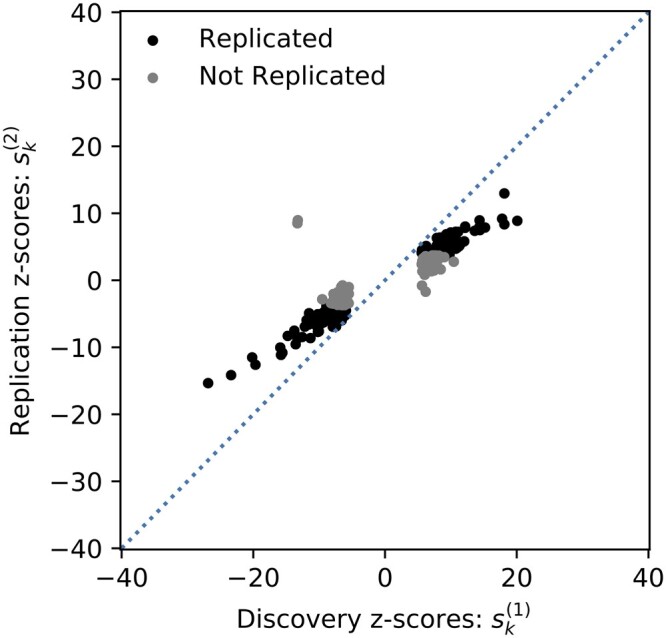
Bivariate GWAS analysis. We perform a bivariate analysis between the z-scores of the discovery and replicate GWAS ($S_{k}^{\left(1\right)}$ and $S_{k}^{\left(2\right)}$, respectively). The significant variants in a discovery GWAS study on height (PMID 25282103) are shown. The variants that replicated successfully using a Bonferonni threshold of 0.05 are shown in black, and the ones that did not replicate are shown in grey. Many variants have stronger associations to the phenotype in the discovery study than the replication study. This phenomenon can be partially explained by WC and partially explained by study-specific confounders. This method jointly models the effects of WC and study-specific confounders on the observed z-scores.

In GWAS, WC is the phenomenon where the association statistics for variants meeting a genome-wide threshold tend to be overestimated. WC can be observed in Fig. 1, where the association statistics for the significant variants in the discovery study are substantially lower in the replication study. Due to this phenomenon, not all of the significant variants in the discovery study replicate. WC is widely observed in GWAS due to lack of statistical power in discovery studies. When power is low, the variants that are most significant in a study are likely to have inflated effect sizes due to random noise.

To model random noise contributing to WC, we model the statistics for each variant *k* from the discovery and replication studies as normally distributed random variables ($s_{k}^{\left(1\right)}$ and $s_{k}^{\left(2\right)}$, respectively). We assume that there is a shared genetic effect *λ* that is responsible for the observed association signal. Thus, the distribution of the statistic for variant *k* in study *i* is $s_{k}^{\left(i\right)}∼N\left(\lambda ,1\right)$. We define the prior probability of the true genetic effect to be $\lambda ∼N(0,\sigma_{g}^{2})$, where the variance in the true genetic effect is learned from the data. Then, we model the joint distribution of the statistics from the 2 studies (Equation 23).

(23) $$\begin{array}{c} \left(\begin{array}{c} s_{k}^{\left(1\right)}\\ s_{k}^{\left(2\right)} \end{array}\right)∼N\left(\left(\begin{array}{c} 0\\ 0 \end{array}\right),\left(\begin{array}{cc} \sigma_{g}^{2}+1 & \sigma_{g}^{2}\\ \sigma_{g}^{2} & \sigma_{g}^{2}+1 \end{array}\right)\right) \end{array}.$$

**Fig. 2.**
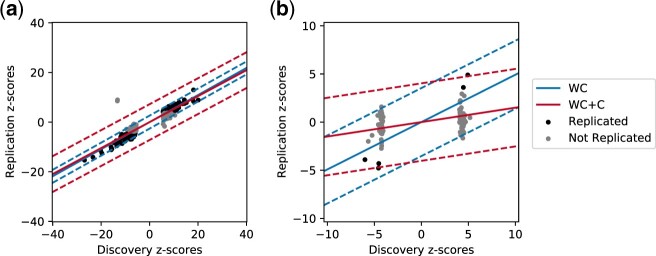
Correcting for WC and confounding. The *x*-axis for each plot is the value of the discovery z-score, and the *y*-axis is the value of the replication z-score. The solid lines correspond to the expected values of the replication z-score given the discovery z-score. The dotted lines represent confidence intervals in the estimates. The blue lines correspond to the model that only accounts for WC, and the red lines correspond to the model that accounts for WC and confounding (WC+C). a) In this GWAS on height (PMID 25282103), there is very little confounding, and a model that accounts for WC explains the majority of the data. b) In this GWAS on height in African American women (PMID 22021425), there is substantial confounding. The model accounting for only WC (blue) does not explain the observed data well, whereas the model with WC and confounding (red) does explain the data well.

We correct for WC by computing the conditional distribution of the replication statistics given the discovery statistics (Equation 24). Using this conditional distribution, we can compute the expected value of the statistic in the replication study, along with confidence intervals on this estimate. This framework accurately models the data in cases where WC is the only source of inflation. Figure 2a shows a GWAS on height (Wood *et al.* 2014), where most of the variants fall within the 95% confidence intervals of the model accounting for WC. This shows that in studies without substantial confounding effects, WC can adequately explain the proportion of variants that replicate or replication rate.

(24) $$(s_{k}^{\left(2\right)}|s_{k}^{\left(1\right)}=x)∼N\left(\frac{\sigma_{g}^{2}}{\sigma_{g}^{2}+1}x,1+\sigma_{g}^{2}-\frac{\sigma_{g}^{4}}{\sigma_{g}^{2}+1}\right).$$

However, there is often additional heterogeneity due to confounding, where a framework that only accounts for WC would not explain the data well. Figure 2b shows an example of a GWAS on height in African American women (Carty *et al.* 2012). In this study, there is substantial confounding, and only 18% of variants replicate. Using a model that only accounts for WC, most variants are outside of the 95% confidence intervals, indicating that there is additional heterogeneity that is not modeled. To account for study-specific confounding, we decompose the effect size of the statistics into a genetic effect (*λ*) and study-specific confounding effects ($\delta^{\left(i\right)}$). The distribution of the statistic for variant *k* in study *i* is $s_{k}^{\left(i\right)}∼N\left(\lambda +\delta^{\left(i\right)},1\right)$. In addition to the prior on the genetic effect, we introduce priors on the study-specific confounders ($\delta^{\left(i\right)}∼N(0,\sigma_{c_{i}}^{2})$). We incorporate both of these priors into the joint distribution of the statistics (Equation 25).

(25) $$\begin{array}{c} \left(\begin{array}{c} s_{k}^{\left(1\right)}\\ s_{k}^{\left(2\right)} \end{array}\right)∼N\left(\left(\begin{array}{c} 0\\ 0 \end{array}\right),\left(\begin{array}{cc} \sigma_{g}^{2}+\sigma_{c_{1}}^{2}+1 & \sigma_{g}^{2}\\ \sigma_{g}^{2} & \sigma_{g}^{2}+\sigma_{c_{2}}^{2}+1 \end{array}\right)\right) \end{array}.$$

We correct for both WC and confounding by computing the conditional distribution of the replication statistic given the discovery statistic (Equation 26). By taking into account the additional variance in the association statistics from confounders, we are able to more accurately model the statistics from the 2 studies (Fig. 2b). We quantitatively assess how well each model fits the data by estimating the number of variants that should replicate under each model (*Methods*). The naive model that only accounts for WC estimated that 56% of variants would replicate, whereas our model that also accounts for confounding estimated that 18% of variants would replicate, which is closer to the observed replication. This difference in the estimated replication under each model is due to the study-specific confounding effects estimated in the second model, which both decreases the expected value of the statistics in the replication study and increases the variance of the statistics in the replication study. After correcting for WC and confounding, most variants are within the 95% confidence intervals for the model. Thus, in this study, modeling study-specific confounders is necessary to explain the observed patterns of replication.

(26) $$(s_{k}^{\left(2\right)}|s_{k}^{\left(1\right)}=x)∼N\left(\frac{\sigma_{g}^{2}}{\sigma_{g}^{2}+\sigma_{c_{1}}^{2}+1}x,\sigma_{g}^{2}+\sigma_{c_{2}}^{2}+1-\frac{\sigma_{g}^{4}}{\sigma_{g}^{2}+\sigma_{c_{1}}^{2}+1}\right).$$

We apply this framework to simulated data and 100 human GWAS in the GWAS catalog. For all data sets, we compute the expected replication rate under the 2 models in order to compare how well each model fits the data, relative to each other.

### WC and confounding accurately explains replication in simulated data

To demonstrate that our approach accurately models the effects of WC and confounding to explain replication rates, we generated simulated data, where the variance in the genetic ($\sigma_{g}^{2}$) and confounding effects ($\sigma_{c1}^{2}$ and $\sigma_{c2}^{2}$) are known. In all simulations, we set the sample size of the discovery study to be 2,000 and the sample size of the replication study to be 1,000. We simulated z-scores for 1 million independent variants in each simulation.

For each simulation, we fixed $\sigma_{g}^{2},\;\sigma_{c1}^{2}$, and $\sigma_{c2}^{2}$ to be one of 4 values and simulated true effect sizes and study-specific confounding effects for each variant. We then simulated z-scores for the 2 studies (*Methods*). For each combination of parameter values, we repeated this 1,000 times to generate a total of 64,000 simulations. We then used a Bonferonni corrected significance threshold of 5e−8 to identify variants that were significant in the discovery study. The number of significant variants in the discovery study for each simulation ranges from 263 to 11,362 variants (Supplementary Fig. 1a). We computed the replication rates as the proportion of variants in the discovery study that met a nominal threshold of 0.05 in the replication study and had the same direction of effect in the 2 studies. The observed replication ranged from 15% to 60%. On average, higher levels of $\sigma_{g}^{2}$ yielded higher replication rates (Supplementary Fig. 1b).

**Fig. 3.**
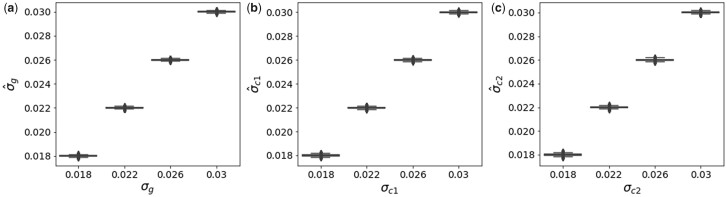
Variance components in WC and confounding simulations. True values of variance components (*x*-axis) vs estimated values (*y*-axis) for a) $\sigma_{g}^{2}$, b) $\sigma_{c1}^{2}$, and c) $\sigma_{c2}^{2}$.

For each simulation, we used the z-scores of variants significant in the discovery study and their corresponding z-scores in the replication study as input to our model. We computed the MLE of $\sigma_{g}^{2},\;\sigma_{c1}^{2}$, and $\sigma_{c2}^{2}$. The estimates of the parameters were accurate and unbiased for all simulations (Fig. 3).

**Fig. 4.**
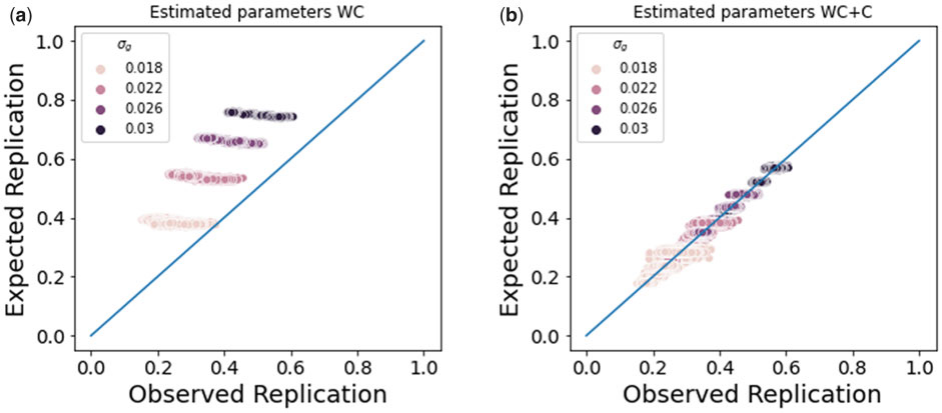
WC and confounding simulations. a) We computed the expected replication rate under the WC model. The WC model over-estimates replication because it does not account for confounding between the studies. b) We computed the expected replication rate under the WC+C model. The WC+C model more accurately describes the observed replication than the WC model.

We used the MLE parameters to compute the expected replication rate under the model and compared this to the observed replication rate to assess the fit of each model. The WC+C more accurately described replication compared to the WC model (Fig. 4, a and b), which we would expect since the simulations have study-specific confounding. In addition, the expected replication rate using the MLE parameters are close to the expected replication rates using the true values of the parameters, indicating that the expected replication rate is robust to variance in the parameter estimates (Supplementary Fig. 2).

### Accounting for missing data in discovery and replication designs

In studies with discovery and replication designs, often only a subset of variants is tested in the replication study. In some studies, only the summary statistics for variants that were significant in the discovery study are reported. The variants that were not significant in the discovery study are missing data. For these missing variants, we compute the likelihood of the data by integrating over all possible values of the data given the significance threshold used in the discovery study (Equation 15).

**Fig. 5.**
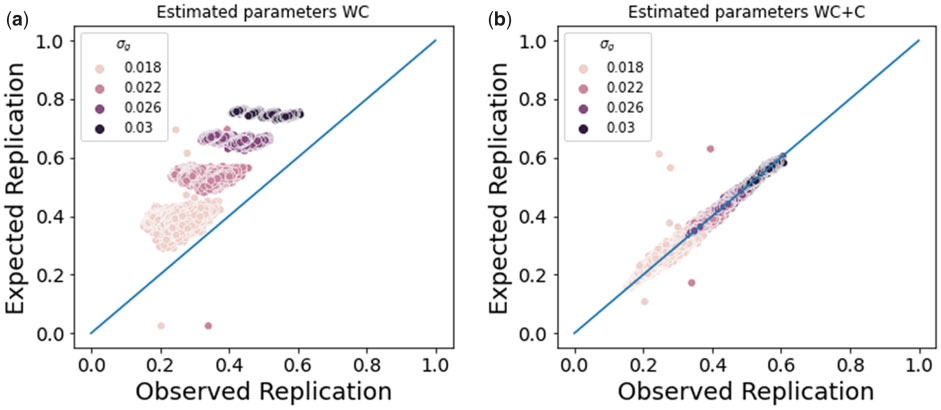
WC and confounding simulations. a) We computed the expected replication rate under the WC model. b) We computed the expected replication rate under the WC+C model.

To evaluate whether the MLE estimates of the parameters are accurate in these situations with missing data, we used the previous set of simulations, where the variance in the genetic ($\sigma_{g}^{2}$) and confounding effects ($\sigma_{c1}^{2}$ and $\sigma_{c2}^{2}$) are known. For this set of analyses, we used the z-scores of the significant variants in the discovery study and their corresponding z-scores in the replication study only to estimate the parameters. The estimates of the parameters were accurate, but the variance in the parameter estimates was higher (Supplementary Fig. 4). Despite the higher variance in the parameter estimates, the expected replication rate using the MLE parameters are close to the expected replication rates using the true values of the parameters, indicating that the expected replication rate is robust to variance in the parameter estimates (Supplementary Fig. 3). Similar to previous simulations, the WC+C more accurately described replication compared to the WC model (Fig. 5, a and b).

### Application to 100 human GWAS datasets

We then applied our framework to 100 human GWAS previously curated to study WC (Palmer and Pe’er 2017). All studies have summary statistic data publicly available, a focus on human genetics, and a discovery and replication design, where only the significant SNPs in the discovery study are tested in the replication study. We used the z-scores from these discovery and replication studies as input to our method and estimated the variance parameters (Supplementary Fig. 5, Supplementary Table 1).

After learning the variance parameters for the genetic and confounding effects, we calculated the estimated replication rates under the 2 models (*Methods*, Fig. 7). We compared these estimated replication rates to the true replication rates to assess which model explained the observed replication better. We defined the true replication rate to be the proportion of variants in the discovery study that are also significant in the replication study with the same direction of effect in both studies. We used a nominal *P*-value threshold of α=0.05 for each replication study. Of the 1,652 reported GWAS variants, only 726 (44%) replicated (Supplementary Table 3). Using the naive model that does not account for confounding, we would expect 973 (56%) of the variants to replicate. However, when we account for both WC and confounding in our framework, we would expect 762 (46%) of the variants to replicate, which is very close to the observed value.

While the naive model that only accounts for WC explained the replication data well in some cases, in others, we observe a substantial bias beyond what we would expect from statistical noise due to WC (Supplementary Fig. 6). The WC and confounding model explained the observed replication well in most studies. Only 5 studies have predicted replication more than 5 SNPs from the true values (Supplementary Fig. 11). We expect discrepancy between the observed and expected replication rates under 2 circumstances. The first is when the number of discovered genome-wide significant variants is low and the variance in the parameter estimates is high, as demonstrated through simulations. The second is when the confounding cannot be accurately modeled by a single linear factor. Since our estimated confounding is study-specific, not variant-specific, more complex patterns of confounding may not be accurately captured by this framework.

**Fig. 6.**
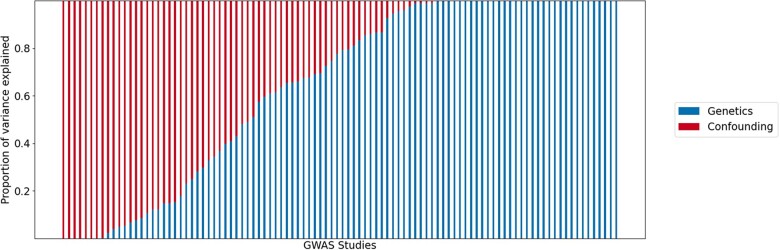
Proportion of variance explained by confounding in 100 human GWAS. Each study is on the *x*-axis. The proportion of variance explained by genetics (blue) and confounding (red) are shown on the y-axis.

We observed a wide range in the estimated values for the variance in the confounding effects, relative to the variance in the genetic effects (Supplementary Fig. 5). To assess the relative contributions of genetics and confounding to replication, we computed the proportion of variance in the discovery z-scores explained by genetics and confounding (*Methods*, Equation 18 and Equation 17). We observed a wide range of estimated confounding levels across the 100 studies (Fig. 6).

**Fig. 7.**
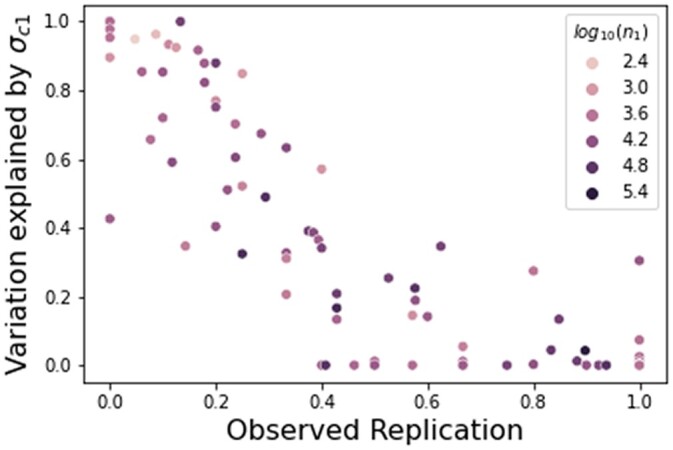
Estimated confounding explains observed replication. The *x*-axis is the observed replication, and the *y*-axis is the proportion of variance in the discovery study explained by confounding. Each dot represents a single GWAS study. The Spearman correlation between the estimated variance of confounding and true replication rate is −0.88. The color corresponds to the number of individuals in the discovery study. While the estimated confounding in the discovery study explains the replication rate well, sample size does not explain the replication consistently.

The proportion of variance explained by confounding for the discovery study was strongly correlated (Spearman ρ=−0.88) with the observed replication, indicating that higher levels of estimated confounding leads to lower replication (Fig. 7). However, sample size was not highly correlated with observed replication (Spearman ρ=0.11) and explained replication inconsistently. While theoretically studies with larger sample sizes tend to have higher power and are more likely to replicate, in practice, some studies with large sample sizes replicate well and others do not. Similarly, some of the smallest studies had the highest replication rates. Another potential cause of poor replication is noise in the measurement of phenotypes. For example, behavioral phenotypes are often more difficult to measure than physiological traits. While the behavioral phenotypes in this analysis tended to have more confounding than physiological traits, the physiological traits had a wide range in observed confounding levels, indicating that type of phenotype measured also cannot fully explain replication patterns (Supplementary Fig. 7).

We attempted to identify confounders that explained the variability in replication across 21 studies with at least 20,000 samples in the discovery cohort (Supplementary Table 2). The replication rate varied widely across these studies (13–94%), despite the relatively large sample sizes. In addition, the variance explained by confounding in the discovery study ranged from 0% to 69%. All of the discovery studies were meta-analyses combining data from multiple cohorts, and all studies corrected association statistics from each cohort using genomic control before combining the data. We first hypothesized that the number of cohorts would adversely affect the replication rate. However, the correlation between the replication rate and the number of cohorts was low (*r* = 0.19, *P*-value = 0.42, Supplementary Table 2). This suggests that with the correct methodology, it is possible to combine data from multiple cohorts without introducing study-specific heterogeneity. We then attempted to see whether the ancestry of the replication data set matched the ancestry of the discovery data set on the continental level. We labeled all discovery studies with multiple ancestries as not having an ancestry matched replication study. The replication rate for studies with ancestry-matched replication studies was not significantly different from the replication rate for studies without ancestry-matched replication studies (Supplementary Fig. 10). While we were unable to determine and factors that explained the variation in replication rate well, there are a number of factors that are difficult to quantify. For example, measurement bias across cohorts depends on both phenotype and study design. PMID 23372041 is a GWAS study on macronutrient intake that had the highest level of confounding estimated (69%) in the 21 studies. In this study, average daily intake of food and nutrients was calculated using data from self-administered questionnaires. In this meta-analysis, 2 cohorts were entirely female, and the other cohort was entirely male. Furthermore, the questionnaires used for the 3 cohorts were different. These 2 differences between cohorts could lead to substantial confounding in the meta-analysis and lack of replication in follow-up studies. One of the benefits of our method is that it can be applied to detect potential confounding without deep understanding of the methodological differences between the discovery and replication cohort.

### Comparison to existing corrections of WC

We compared our estimated replication rates under the WC model with those previously reported in Palmer and Pe’er (2017), which corrected for WC using a previously published method, which we refer to as “ZhongPrentice” (Zhong and Prentice 2008). At a nominal significance level of 0.05 for the replication study, Palmer *et al.* estimated that 888 loci would replicate, which is more than the observed replication rate (726 variants). However, it is substantially closer to the observed replication rate than our WC only model, which estimated that 973 variants would replicate.

The primary difference between our estimated replication under the naive WC model and the estimated replication using ZhongPrentice is that our framework model’s WC by accounting for uncertainty in the true effect sizes of the variants. ZhongPrentice treats the true effect size as fixed and attempts to estimate the true effect size by removing the bias due to WC, which is modeled as a function of the true effect and the significance threshold for the discovery study. In this framework, variants with true effect sizes close to the significance threshold of the discovery study have high bias due to WC, regardless of whether the estimated effect size was inflated or not.

In practice, the true effect size is not known, so it is difficult to compare these WC corrections in real data. To compare these 2 models of WC, we simulated GWAS z-scores for discovery and replication cohorts, where the true effect size was known and the study-specific confounding was set to zero. For each simulation, we fixed *σ_g_* to be one of 4 values, and we fixed $\sigma_{c1}=0$ and $\sigma_{c2}=0$. We simulated z-scores for 1 million independent variants in each simulation. For each combination of parameter values, we repeated this simulation procedure 1,000 times to generate a total of 4,000 simulations. We then used a Bonferonni threshold of 5e−8 to identify variants that were significant in the discovery study. The number of significant variants in the discovery study for each simulation ranges from 0 to 1,234 variants (Supplementary Fig. 8a). We computed the replication rates as the proportion of variants in the discovery study that met a nominal threshold of 0.05 in the discovery study and had the same direction of effect in the 2 studies. The observed replication ranged from 0% to 84% (Supplementary Fig. 8b.

**Fig. 8.**
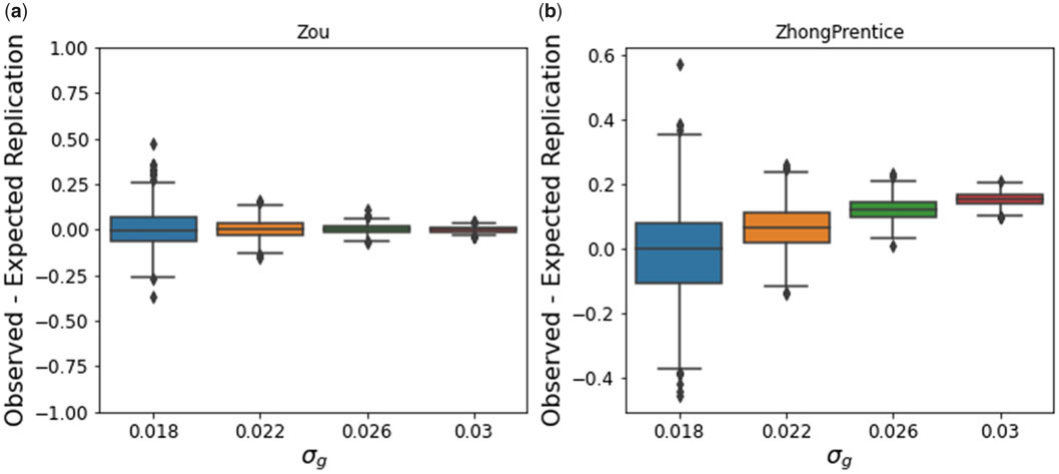
Comparison of methods accounting for WC. We simulated discovery and replication z-scores without study-specific confounding effects for a range of *σ_g_* values. The difference between the observed and expected replication under a) our method. B) ZhongPrentice method.

We computed the MLE estimates of the variance components. For all simulations, the estimation of the parameters was accurate (Supplementary Fig. 9). We computed the difference in the observed and expected replication rates after accounting for WC under our model and ZhongPrentice. As *σ_g_* increases, the difference between the observed and expected replication rates decreases on average for both models (Fig. 8). While our method is unbiased for all values of *σ_g_*, ZhongPrentice underpredicts the observed replication as *σ_g_* increases (Fig. 8).

## Discussion

We developed a novel statistical framework to correct for WC and study-specific confounding in GWAS data. This framework utilizes GWAS replications to identify the presence of confounders without relying on assumptions to distinguish between polygenicity and confounding.

We showed through simulations that our model that accounts for WC and Confounding provides accurate estimates of the expected replication rate, even when using incomplete data in a discovery and replication data set. However, the variance in the estimates is higher when using incomplete data to estimate the variance parameters. Thus, if z-scores for variants that are not genome-wide significant are available, it is best to include those variants when estimating the parameters.

When applying our method to 100 human GWAS, we showed that a model that accounts for WC and confounding explains replication rates more accurately than a naive model that only accounts for WC. We observed a range of confounding levels in the 100 GWAS studies analyzed and showed that estimated variance explained by confounding in the discovery study explains the observed replication across studies well and that it can be used to identify studies that fail to replicate due to confounding between the discovery and replication cohorts.

One limitation of our framework is that our estimate of variance explained by confounding in the discovery study does not explain the source of the confounding. In order to better understand the sources of confounding in the 100 human GWAS, we conducted more thorough analysis of 21 GWAS with at least 20,000 samples in the discovery study. While factors such as sample size, type of phenotype, number of cohorts, ancestry, and heterogeneity correction methodology inconsistently explained replication rate in these data, we made several observations with interesting implications. First of all, correcting statistics from each cohort of a meta-analysis using genomic control may not be sufficient to control for study-specific heterogeneity. In our analysis of 21 meta-analyses with at least 20,000 samples in the discovery study, all studies used genomic control. However, estimated variation explained by confounding from the discovery study ranged from 0% to 69%. Second, a few meta-analysis studies performing additional analyses to check for residual differences between cohorts tended to have better replication. For example, PMID 25282103 had high replication (90%) and low confounding (4%). This study validated their findings using a linear model with a linear mixed model. This analysis provided support that the inflated statistics were due to the polygenic nature of the trait, rather than residual ancestry differences (Wood *et al.* 2014). Exploring potential sources of confounding and validating that these factors do not drive associations in the discovery phase is the gold standard for association analysis and critical for high replication in follow-up studies. While exploring all potential sources of confounding between studies is the gold standard, this process is often time-consuming and difficult if raw data and meta-data are not available.

One application of this framework would be to identify which studies to include in a meta-analysis or mega-analysis. In GWAS, meta-analysis has discovered many associations that were not identified by each individual study (Lam *et al.* 2017; Nagel *et al.* 2018). However, if confounding exists between studies, novel variants found when combining the data could be false positives. It has been proposed to only apply meta-analysis between GWAS that have a high genetic correlation (*rG* > 0.7; Okbay *et al.* 2016; Fontana *et al.* 2018). However, it has also been observed that studies can have confounding and poor replication despite high genetic correlation (Zhou *et al.* 2019). Our method can be used to determine whether study-specific heterogeneity due to confounders exists before combining data from independent cohorts. While our current framework can only be used for pairs of studies, when combining multiple studies, a pairwise analysis could be performed by designating 1 data set as the discovery set and comparing this dataset with each of the other datasets. Alternatively, our framework could be extended to model an arbitrary number of studies by replacing the joint distribution of z-scores from the discovery and replication study with a multivariate normal distribution of z-scores from all studies.

Our method uses summary statistics to quickly determine why studies fail to replicate and quantify confounding between studies. As more association studies are performed, the outstanding challenge is to integrate these data sets. We expect that this method will be useful for better understanding why studies fail to replicate and for determining whether to combine data from data from independent cohorts.

## Supplementary Material

jkac261_Supplementary_Figure_S1

jkac261_Supplementary_Figure_S2

jkac261_Supplementary_Figure_S3

jkac261_Supplementary_Figure_S4

jkac261_Supplementary_Figure_S5

jkac261_Supplementary_Figure_S6

jkac261_Supplementary_Figure_S7

jkac261_Supplementary_Figure_S8

jkac261_Supplementary_Figure_S9

jkac261_Supplementary_Figure_S10

jkac261_Supplementary_Figure_S11

jkac261_Supplemental_Material

jkac261_Supplementary_Table_S1

jkac261_Supplementary_Table_S2

jkac261_Supplementary_Table_S3

## Data Availability

Supplemental material is available at G3 online.

## References

[jkac261-B1] Bulik-Sullivan B , Finucane HK, Anttila V, Gusev A, Day FR, Duncan L, Perry JR, Patterson N, Robinson EB, Daly MJ, et al; ReproGen Consortium; Psychiatric Genomics Consortium; Genetic Consortium for Anorexia Nervosa of the Wellcome Trust Case Control Consortium 3. An atlas of genetic correlations across human diseases and traits. bioRxiv. 2015;47:1–44. 10.1038/ng.3406 PMC4797329 26414676

[jkac261-B2] Carty CL , Johnson NA, Hutter CM, Reiner AP, Peters U, Tang H, Kooperberg C. Genome-wide association study of body height in African Americans: the Women ’ s Health Initiative SNP Health Association Resource (SHARe). Hum Mol Genet. 2012;21(3):711–720. 22021425 10.1093/hmg/ddr489 PMC3259012

[jkac261-B3] de Vlaming R , Johannesson M, Magnusson PKE, Ikram MA, Visscher PM. Equivalence of LD-score regression and individual-level-data methods. bioRxiv. 2017;211821. https://doi.org/10.1101/211821.

[jkac261-B4] Devlin B , Roeder K. Genomic control for association studies. Biometrics. 1999;55(4):997–1004. 11315092 10.1111/j.0006-341x.1999.00997.x

[jkac261-B5] Eskin E. Discovering genes involved in disease and the mystery of missing heritability. Commun ACM. 2015;58(10):80–87.

[jkac261-B6] Greene CS , Penrod NM, Williams SM, Moore JH. Failure to replicate a genetic association may provide important clues about genetic architecture. PLoS One. 2009;4(6):e5639. 19503614 10.1371/journal.pone.0005639 PMC2685469

[jkac261-B7] Ioannidis JPA. Why most clinical research is not useful. PLoS Med. 2016;13(6):e1002049. 27328301 10.1371/journal.pmed.1002049 PMC4915619

[jkac261-B8] Ioannidis JPA. Why most published research findings are false. PLoS Med. 2005;2(8):e124. 16060722 10.1371/journal.pmed.0020124 PMC1182327

[jkac261-B9] Joo JWJ , Sul JH, Han B, Ye C, Eskin E. Effectively identifying regulatory hotspots while capturing expression heterogeneity in gene expression studies. Genome Biol. 2014;15(4):r61. 24708878 10.1186/gb-2014-15-4-r61 PMC4053820

[jkac261-B10] Kang HM , Ye C, Eskin E. Accurate discovery of expression quantitative trait loci under confounding from spurious and genuine regulatory hotspots. Genetics. 2008;180(4):1909–1925. 18791227 10.1534/genetics.108.094201 PMC2600931

[jkac261-B11] Lam M , Trampush JW, Yu J, Glahn DC, Malhotra AK, Lam M, Trampush JW, Yu J, Knowles E, Davies G, et al Large-scale cognitive GWAS meta-analysis reveals tissue-specific neural expression and potential nootropic drug targets resource large-scale cognitive GWAS meta-analysis reveals tissue-specific neural expression and potential nootropic drug targets. Cell Rep. 2017;21(9):2597–2613. 29186694 10.1016/j.celrep.2017.11.028 PMC5789458

[jkac261-B12] Marigorta UM , Rodríguez JA, Gibson G, Navarro A. Replicability and prediction: lessons and challenges from GWAS. Trends Genet. 2018;34(7):504–517. 29716745 10.1016/j.tig.2018.03.005 PMC6003860

[jkac261-B13] Nagel M , Jansen PR, Stringer S, Watanabe K, Leeuw CAD, Bryois J, Savage JE, Hammerschlag AR, Skene NG, Mun AB, et al; 23andMe Research Team. Meta-analysis of genome-wide association studies for neuroticism in 449, 484 individuals identifies novel genetic loci and pathways. Nat Genet. 2018;50(7):920–927. 29942085 10.1038/s41588-018-0151-7

[jkac261-B14] Okbay A , Baselmans BML, De Neve J-E, Turley P, Nivard MG, Fontana MA, Meddens SFW, Linnér RK, Rietveld CA, Derringer J, et al; LifeLines Cohort Study. Genetic variants associated with subjective well-being, depressive symptoms and neuroticism identified through genome-wide analyses. Nat Genet. 2016;48(6):624–633. 27089181 10.1038/ng.3552 PMC4884152

[jkac261-B15] Palmer C , Pe’er I. Statistical correction of the Winner’s Curse explains replication variability in quantitative trait genome-wide association studies. PLoS Genet. 2017;13(7):e1006916. 28715421 10.1371/journal.pgen.1006916 PMC5536394

[jkac261-B16] Patil P , Bachant-Winner PO, Haibe-Kains B, Leek JT. Test set bias affects reproducibility of gene signatures. Bioinformatics. 2015;31(14):2318–2323. 25788628 10.1093/bioinformatics/btv157 PMC4495301

[jkac261-B17] Patterson N , Price AL, Reich D. Population structure and eigenanalysis. PLoS Genet. 2006;2(12):e190. 17194218 10.1371/journal.pgen.0020190 PMC1713260

[jkac261-B18] Price AL , Patterson NJ, Plenge RM, Weinblatt ME, Shadick NA, Reich D. Principal components analysis corrects for stratification in genome-wide association studies. Nat Genet. 2006;38(8):904–909. 16862161 10.1038/ng1847

[jkac261-B19] Stegle O , Parts L, Piipari M, Winn J, Durbin R. Using Probabilistic Estimation of Expression Residuals (PEER) to obtain increased power and interpretability of gene expression analyses. Nat Protoc. 2012;7(3):500–507. 22343431 10.1038/nprot.2011.457 PMC3398141

[jkac261-B20] Sun L , Dimitromanolakis A, Faye LL, Paterson AD, Waggott D, Bull SB; DCCT/EDIC Research Group. BR-squared: a practical solution to the winner’s curse in genome-wide scans. Hum Genet. 2011;129(5):545–552. 21246217 10.1007/s00439-011-0948-2 PMC3074069

[jkac261-B21] Turley P , Walters RK, Maghzian O, Okbay A, Lee JJ, Fontana MA, Nguyen-Viet TA, Wedow R, Zacher M, Furlotte NA, et al Social Science Genetic Association Consortium. Multi-trait analysis of genome-wide association summary statistics using MTAG Patrick. Nat Genet. 2018;50(2):229–237. 29292387 10.1038/s41588-017-0009-4 PMC5805593

[jkac261-B22] Welter D , MacArthur J, Morales J, Burdett T, Hall P, Junkins H, Klemm A, Flicek P, Manolio T, Hindorff L, et al The NHGRI GWAS Catalog, a curated resource of SNP-trait associations. Nucleic Acids Res. 2014;42(D1):D1001–D1006. 24316577 10.1093/nar/gkt1229 PMC3965119

[jkac261-B23] Wood AR , Esko T, Yang J, Vedantam S, Pers TH, Gustafsson S, Chu AY, Estrada K, Luan J, Kutalik Z, et al; LifeLines Cohort Study. Defining the role of common variation in the genomic and biological architecture of adult human height. Nat Genet. 2014;46(11):1173–1186. 25282103 10.1038/ng.3097 PMC4250049

[jkac261-B24] Xiao R , Boehnke M. Quantifying and correcting for the winner’s curse in genetic association studies. Genet Epidemiol. 2009;33(5):453–462. 19140131 10.1002/gepi.20398 PMC2706290

[jkac261-B25] Xiao R , Boehnke M. Quantifying and correcting for the winner’s curse in quantitative trait association studies. Genet Epidemiol. 2011;35(3):133–138. 21284035 10.1002/gepi.20551 PMC3500533

[jkac261-B26] Xu S. Theoretical basis of the Beavis effect. Genetics. 2003;165(4):2259–2268. 14704201 10.1093/genetics/165.4.2259 PMC1462909

[jkac261-B27] Zhong H , Prentice RL. Bias-reduced estimators and confidence intervals for odds ratios in genome-wide association studies. Biostatistics. 2008;9(4):621–634. 18310059 10.1093/biostatistics/kxn001 PMC2536726

[jkac261-B28] Zhong H , Prentice RL. Correcting “winner’s curse” in odds ratios from genomewide association findings for major complex human diseases. Genet Epidemiol. 2010;34(1):78–91. 19639606 10.1002/gepi.20437 PMC2796696

[jkac261-B29] Zhou X St. , Pierre CL, Gonzales NM, Cheng R, Chitre A, Sokoloff G, Palmer AA. Genome-wide association study in 2 cohorts from a multi-generational mouse advanced intercross line highlights the difficulty of replication. bioRxiv. 2019. https://doi.org/10.1534/g3.119.400763. 10.1534/g3.119.400763 PMC7056977 31974095

