# Supplementary figures and images for: Accurate modeling of replication rates in genome-wide association studies by accounting for Winner’s Curse and study-specific heterogeneity
Source: G3 (Bethesda). 2022 Oct 17;12(12):jkac261. doi: 10.1093/g3journal/jkac261 (PMC9713380; doi:10.1093/g3journal/jkac261)

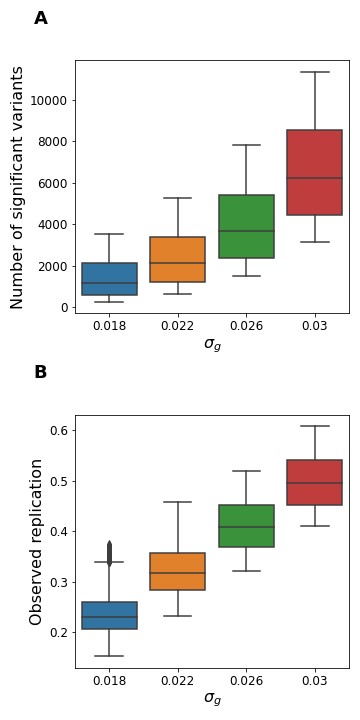

Supplement: jkac261_Supplementary_Figure_S1 [file jkac261_supplementary_figure_s1.jpeg]

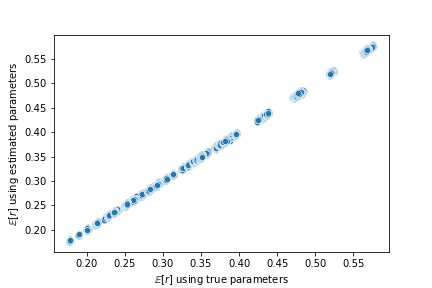

Supplement: jkac261_Supplementary_Figure_S2 [file jkac261_supplementary_figure_s2.jpeg]

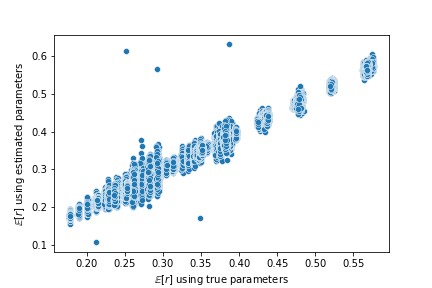

Supplement: jkac261_Supplementary_Figure_S3 [file jkac261_supplementary_figure_s3.jpeg]

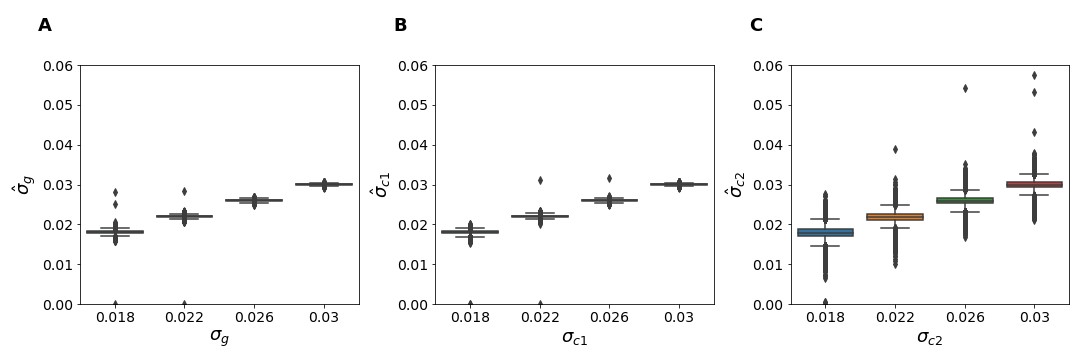

Supplement: jkac261_Supplementary_Figure_S4 [file jkac261_supplementary_figure_s4.jpeg]

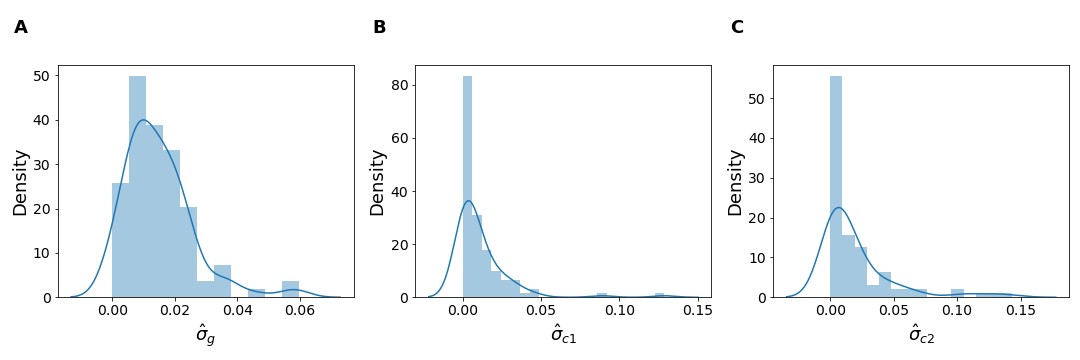

Supplement: jkac261_Supplementary_Figure_S5 [file jkac261_supplementary_figure_s5.jpeg]

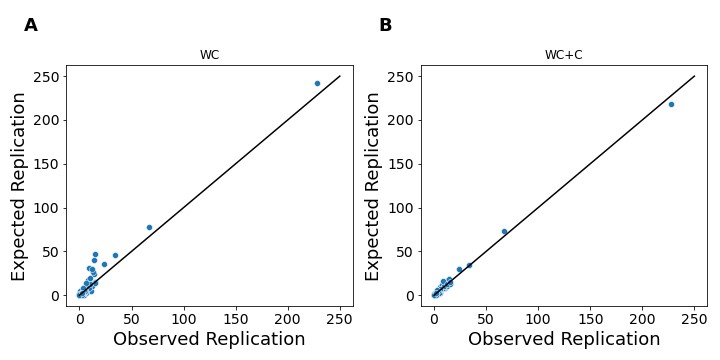

Supplement: jkac261_Supplementary_Figure_S6 [file jkac261_supplementary_figure_s6.jpeg]

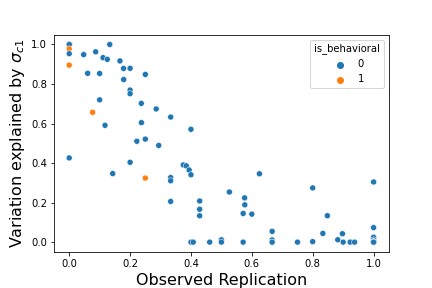

Supplement: jkac261_Supplementary_Figure_S7 [file jkac261_supplementary_figure_s7.jpeg]

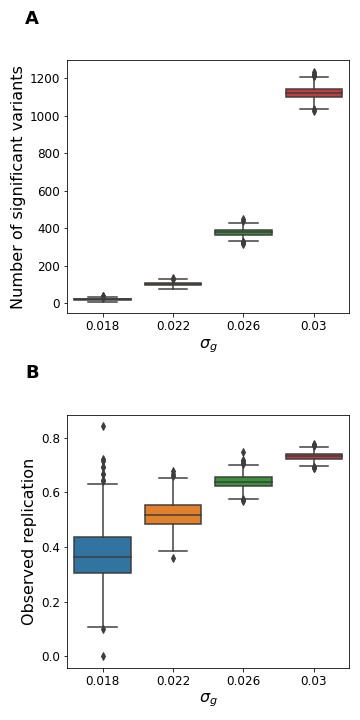

Supplement: jkac261_Supplementary_Figure_S8 [file jkac261_supplementary_figure_s8.jpeg]

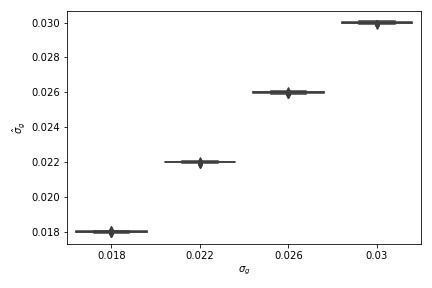

Supplement: jkac261_Supplementary_Figure_S9 [file jkac261_supplementary_figure_s9.jpeg]

$t = -1.27, p = .21$

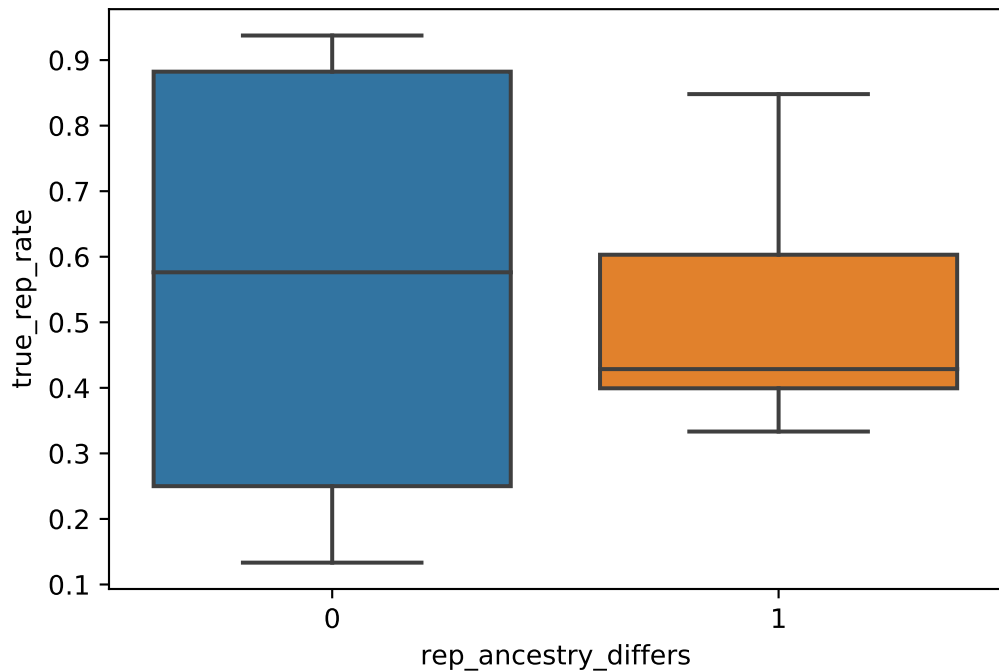

Supplement: jkac261_Supplementary_Figure_S10 [file jkac261_supplementary_figure_s10.pdf]

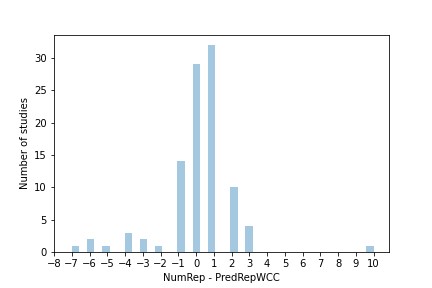

Supplement: jkac261_Supplementary_Figure_S11 [file jkac261_supplementary_figure_s11.jpeg]
