# Supplementary material for: Accurate modeling of replication rates in genome-wide association studies by accounting for Winner’s Curse and study-specific heterogeneity
Source: G3 (Bethesda). 2022 Oct 17;12(12):jkac261. doi: 10.1093/g3journal/jkac261 (PMC9713380; doi:10.1093/g3journal/jkac261)
Supplement: jkac261_Supplemental_Material [file jkac261_supplemental_material.pdf]

Supplementary Materials

Table S1: **Application to 100 human GWAS** We applied our method 100 human GWAS data sets previously published in the articles referenced by PMID. The sample size of the discovery study is “n1” and the sample size of the replication study is “n2”. The significance threshold used in the discovery study is “t”, and the replication threshold is 0.05. The estimated values of the parameters are “sigma g”, “sigma c1”, and “sigma c2”. The total number of variants significant in the discovery study is “num sig”. The number of variants that replicated is “num rep”. The number of variants that are expected to replicate under the WC and WC+C models are “rep wc” and “rep wcc”, respectively. The proportion of variance explained by genetics and confounding are “var exp g” and “var exp c1”, respectively. An indicator variable (“is behavioral”) indicates whether the phenotype is behavioral or not.

Table S2: **Human GWAS meta-analyses** We applied our method 21 human GWAS meta-analyses previously published in the articles referenced by PMID. The sample size of the discovery study is “n1” and the sample size of the replication study is “n2”. All studies had discovery sample size greater than 20,000. The significance threshold used in the discovery study is “t”, and the replication threshold is 0.05. The estimated values of the parameters are “sigma g”, “sigma c1”, and “sigma c2”. The total number of variants significant in the discovery study is “num sig”. The number of variants that replicated is “num rep”. The number of variants that are expected to replicate under the WC and WC+C models are “rep wc” and “rep wcc”, respectively. The proportion of variance explained by genetics and confounding are “var exp g” and “var exp c1”, respectively. “is behavioral” indicates whether the phenotype is behavioral or not. The number of cohorts in the meta-analysis is shown in “num cohorts”. “rep ancestry differs” indicates whether the replication study’s ancestry differs from the discovery study’s ancestry on a continental level.

Table S3: **Summary statistics from 100 human GWAS** For each variant that was significant in the discovery study, we computed z-scores for the discovery study (“s1”) as the effect size (“beta.disc”) divided by the standard error (“se.disc”). We also computed the corresponding z-scores for the replication study (“s2”) as the effect size (”beta.rep”) divided by the standard error (“se.rep”). The SNP can be identified using the RSID (“rsid”), the chromosome (“chr”), and the position (“pos”). The phenotype can be identified by the PubMed ID (”file.tag”). The “is.rep” column contains a 1 if the variant passed the nominal replication threshold and contains a 0 if it did not replicate.

Figure S1: **Summary of simulated data** A) Number of significant variants in the discovery study for Winner's Curse and confounding simulations. We fixed the values of the variance parameters and simulated z-scores for discovery and replication cohorts. The x-axis corresponds to the value of σ_g used to generate the simulations, and the y-axis corresponds to the number of significant variants in the discovery study using a Bonferroni threshold of 5e-8. B) Replication in Winner's Curse and confounding simulations. We computed the replication rates as the proportion of variants significant in the discovery study that met a replication threshold of 0.05 in the replication study and had the same direction of effect in the two studies.

Figure S2: **Expected replication rate is robust to variance in MLE parameter estimates.** We computed the expected replication rate using the MLE parameter estimates (y-axis) and compared this to the expected replication rate using the true parameters (x-axis). The expected replication using the two sets of parameters are nearly identical, indicating that the parameters are estimated accurately.

Figure S3: **Expected replication rate is robust to variance in MLE parameter estimates with missing data.** We computed the expected replication rate using the MLE parameter estimates (y-axis) and compared this to the expected replication rate using the true parameters (x-axis). Despite the increased variance in the parameter estimates when using incomplete data, the expected replication with MLE parameters is similar to expected replication using true parameters.

Figure S4: **Variance components in Winner's Curse and Confounding simulations with incomplete data.** True values of variance components (x-axis) vs estimated values (y-axis) for A) σ_g^2 B) σ_{c1}^2 C) σ_{c2}^2

Figure S5: **Distribution of variance components in 100 human GWAS.** Distribution of MLE estimates of A) σ_g B) σ_{c1} c)σ_{c2}

Figure S6: **Expected replication in 100 human GWAS.** The x-axis is the true number of variants that replicate. The y-axis is the estimated number of variants that replicate under a model. Each dot represents one GWAS study. A) Expected replication under WC model B) Expected Replication under WC+C model

Figure S7: **Behavioral phenotypes have higher levels of confounding.** The x-axis is the MLE of $\sigma_{c_1}^2$, and the y-axis is the true replication rate. Each dot represents a single GWAS study. The color corresponds to whether the phenotype is behavioral or not. Behavioral phenotypes tend to have higher estimated levels of confounding.

Figure S8: **Summary of simulated data for Winner's Curse comparisons** A) Number of significant variants in the discovery study for Winner's Curse simulations. We fixed the values of the variance parameters and simulated z-scores for discovery and replication cohorts. The x-axis corresponds to the value of σ_g used to generate the simulations, and the y-axis corresponds to the number of significant variants in the discovery study using a Bonferroni threshold of 5e-8. B) Replication in Winner's Curse and confounding simulations. We computed the replication rates as the proportion of variants in the discovery study that met a threshold of 0.05 in the replication study and had the same direction of effect in the two studies.

Figure S9: **Variance Components in Winner's Curse Simulations.** True values of variance components (x-axis) vs estimated values (y-axis) for σ_g^2

Figure S10: **Different replication ancestry fails to explain variation in replication rate.** We identified studies with at least 20,000 individuals in the discovery study. These studies have relatively large sample sizes, yet there is a range of replication rates. We compared the replication rates (y-axis) of studies with the same ancestries between the discovery and replication cohorts (x-axis = 0) and those with different ancestries between the cohorts (x-axis = 1). The replication rates were not significantly different between these two categories of studies (t-test, t = -1.27, p = .21)

Figure S11: **Difference in true and predicted replication in human GWAS.** For the 100 human GWAS, we computed the difference in the number of variants that replicated and the number predicted to replicate under the Winner's Curse and Confounding model (NumRep - PredRepWCC). The distribution is centered around zero, and only five studies have a predicted replication of more than 5 SNPs from the true replication.
